# Co-occurrence of Anti-GAD65 Syndrome, Type 1 Diabetes Mellitus, and Focal Seizures With Impaired Awareness

**DOI:** 10.7759/cureus.40611

**Published:** 2023-06-18

**Authors:** Kieu-Tram Bach, Shahini Ananth, Ikjot Thind, Ning Zhong, Forshing Lui

**Affiliations:** 1 Clinical Sciences, California Northstate University College of Medicine, Sacramento, USA; 2 Neurology, Kaiser Permanente Sacramento Medical Center, Sacramento, USA; 3 Clinical Sciences, California Northstate University College of Medicine, Elk Grove, USA

**Keywords:** muscle spasm, type 1 diabetes mellitus (t1d), neuro-immunology, focal seizure, anti-gad antibody

## Abstract

Glutamic acid decarboxylase (GAD) is an intracellular enzyme found in the presynaptic end of nerve terminals that functions to synthesize ​​gamma-aminobutyric acid (GABA) via decarboxylation. Autoantibodies to the GAD65 isoform have been found in high levels in neurological disorders including stiff person syndrome (SPS), autoimmune encephalitis, and refractory epilepsy. Low levels of anti-GAD65 have also been noted in type 1 diabetes mellitus. We present the unusual case of a woman with a longstanding history of focal seizures with impaired awareness and type 1 diabetes mellitus who was found to have extremely high titers of anti-GAD65 and clinical presentation suggestive of stiff person syndrome. This case highlights the increasing significance of autoimmune etiologies within neurologic disorders, as well as the importance of maintaining a high index of suspicion for rare anti-GAD65 syndromes. Although uncommon and with an unclear pathophysiology, we discuss the importance of establishing SPS diagnostic criteria to facilitate timely diagnosis and quickly direct patient management towards immunotherapy.

## Introduction

Glutamic acid decarboxylase (GAD) is an enzyme expressed in neurons involved in the rate-limiting step of gamma-aminobutyric acid (GABA) synthesis, the primary inhibitory neurotransmitter within the central nervous system [1]. Two isoforms of GAD exist, GAD65 and GAD67, which differ in molecular composition, localization, and activity [1]. While GAD65 is primarily expressed at presynaptic end terminals of nerves, GAD67 can be found diffusely in the cell body and dendrites [2]. GAD67 activity is constant and responsible for basal levels of GABA, while GAD65 exists most commonly in an inactive form and can be activated via binding to cofactor pyridoxal 5’-phosphate (PLP) for rapid GABA synthesis [3].

Antibodies to GAD65 (GAD65 Ab) have been associated with immune-mediated neurological disorders such as stiff person syndrome (SPS), autoimmune limbic encephalitis (LE), cerebellar ataxia (CA), and temporal lobe epilepsy (TLE). GAD65 Ab is hypothesized to inhibit the action of GAD65, thereby blocking GABA synthesis and inducing a state of neuronal hyperexcitability [3]. This mechanism may explain the simultaneous contraction of agonist and antagonist muscles seen in SPS. Additionally, GAD65 expression can be found in pancreatic beta cells and mildly elevated levels of GAD65 Ab have been noted in up to 80% of patients with type 1 diabetes mellitus (DM1) [4]. Typically, GAD65 Ab titers are significantly higher in relation to neurological syndromes compared to DM1, and only GAD-related neurological syndromes will have positive GAD65 Ab in cerebrospinal fluid (CSF); no significant differences were noted in GAD65 Ab titers between SPS, LE, or CA [5]. This suggests that GAD65 Ab may not be directly involved in the pathophysiology of GAD-related neurological syndromes. Furthermore, a correlation between serum or CSF GAD65 Ab with severity, duration, or response to immunotherapy has not been well-established [6, 7].

We present an unusual case of a woman with a complicated history of focal seizures with impaired awareness, neuromuscular disorder, and adult-onset type 1 diabetes mellitus who was found to have extremely high titers of anti-GAD65 and clinical presentation suggestive of stiff person syndrome. 

## Case presentation

The patient is a 46-year-old African American woman who has a significant history of seizures and neuromuscular issues, which will be discussed in more depth. Additionally, her medical records reveal a type I diabetes mellitus diagnosis at age 28, for which she undergoes treatment with an insulin pump. Complications of her diabetes include peripheral neuropathy, bilateral moderate nonproliferative retinopathy with macular edema, stage 3B chronic kidney disease (glomerular filtration rate [GFR] 30-44), and gastroparesis. Furthermore, the patient has received diagnoses of thrombophlebitis affecting the right superficial arm vein, secondary hyperparathyroidism due to chronic kidney disease, iron deficiency anemia, and dysphagia.

Seizure history

The patient first presented to her physician in 1996 at the age of 19 following sporadic occurrences of generalized tonic-clonic (GTC) seizures. At the age of 33 in 2010, the patient developed focal seizures with impaired awareness; during these episodes, the patient reported experiencing a tingling sensation and tightness encompassing her body as well as visual and auditory hallucinations. Per bystanders, she was not responsive when these events occurred. During a follow-up visit, she also reported the following symptoms: déjà vu feeling, hearing similar words, flushing and pain, and a sensation of “everything falling apart”. She would clench up with no apparent loss of consciousness while being able to see/hear individuals conversing but with a lack of comprehension. These episodes lasted 1-2 min with immense postictal fatigue alleviated by hours-long rest. The patient continues to experience sporadic focal seizures with impaired awareness; however, no recurrent GTC seizures have been reported.

The patient has tried the following medications: topiramate, which was ineffective; oxcarbazepine, which was initially effective but lost its efficacy; lamotrigine, which proved effective with no loss in efficacy; pregabalin, utilized for pain rather than seizure.

Neuromuscular history

Since 2010, the patient recalls the beginnings of joint pain as well as bursitis and arthritis of the hips. She has gradually felt the loss of muscle function with the inability to stand for long periods of time. The patient occasionally feels her hips lock, spasming of the thighs which sometimes extends to the whole body, and loss of balance. This has progressed to the patient experiencing some falls because of these symptoms. She indicates that a significant trigger for her muscle spasm is when she feels startled. Alleviating factors include changing position and medications such as pregabalin and tizanidine. Selected diagnostic reports and lab values are summarized in Table 1.

**Table 1:** Summary of the patient’s clinical presentation and diagnostic studies stratified by the patient's age and the year of presentation. GTC: generalized tonic-clonic seizures; HbA1C: glycated hemoglobin; GFR: glomerular filtration rate; anti-GAD Ab: anti-glutamic acid decarboxylase antibodies; anti-dsDNA: anti-double stranded DNA; EMG: electromyogram

Age (year)	Clinical presentation	Diagnostic studies
19 (1996)	Occasional GTC seizures	
28 (2005)	Type I Diabetes, with complications of peripheral neuropathy, nephropathy, and retinopathy	HbA1C 8.4%. GFR: 30-44 ml/L. Anti-GAD Ab: >250
33 (2010)	Focal seizures with impaired awareness, preceded by aura of déju vu. Responded to treatment with lamotrigine after failures with topiramate and oxcarbazepine	EEG: Interictal and ictal activities in temporal and occipital regions of the brain
33-44 (2010-21)	Bursitis and arthritis of hips. Progressive loss of muscle function with spasms and falls. Muscle spasms triggered by startle, and muscle relaxants	Rheumatoid factor, anti-dsDNA, anti-Smith: all negative. Anti-GAD Ab: >250. EMG: axonal sensorimotor peripheral neuropathy. Brain and cervical spine MRI are negative.

## Discussion

Here we reported a case centered on a 46-year-old female with a past medical history of Type 1 diabetes and focal seizures with impaired awareness, who presented with symptoms consistent with stiff person syndrome (SPS), and anti-GAD65 titers superseding maximum levels detected by laboratory measurements. This case highlights the increasing significance of autoimmune etiologies in neurological syndromes such as SPS. What makes this case unique is the onset and diagnosis of type 1 diabetes at an older age as well as the markedly elevated titers of anti-GAD65. Further, the patient’s history of focal seizures with impaired awareness contributes to the growing body of literature that connects anti-GAD65 to a wide spectrum of neurological diseases.

Stiff person syndrome and anti-GAD65 

The diagnosis of stiff person syndrome, formally introduced in 1956, comprises a broad range of symptoms [8]. However, the classic clinical features include painful episodic spasms that can arise spontaneously or are associated with triggers such as loud sounds, and proximal muscle rigidity. Mechanistically, the stiffness is thought to be the combined effect of simultaneously activating agonist and antagonist muscles [8,9,10]. This can ultimately result in postural instability, falls, and issues with ambulation, as seen in our patient [10]. With regard to etiology, it has been purported that SPS is due to decreased GABAergic neurotransmission and subsequent neuronal excitation [11]. This is supported by a strong association with autoimmune diseases such as type 1 dm and vitiligo, and high antibody titers of anti-glutamic acid decarboxylase 65 (Anti-GAD65), an antibody against the rate-limiting enzyme responsible for GABA synthesis [9,11,12]. 

Studies analyzing the association between Anti-GAD65 levels and clinical diagnosis and presentation reveal interesting findings. It has been purported that higher titers of anti-GAD65 can be used as a diagnostic proxy with some studies even suggesting it be pathognomonic for syndromes such as SPS [4,12,13]. Despite this certainty, higher titers are not associated with clinical severity and overall course [14]. However, establishing an anti-GAD65 threshold for diagnosis remains crucial, especially in cases such as our patient’s. Despite our patient presenting with classical clinical symptoms and DM1, our patient’s electromyogram (EMG)findings were unremarkable with no evidence of abnormal motor units. This poses a diagnostic challenge as there is no objective laboratory finding except for the presence of extremely elevated titers of anti-GAD65. According to some, the combination of anti-GAD65 and clinical presentation alone may be sufficient to diagnose SPS [13].

Type 1 diabetes mellitus and stiff person syndrome

Additionally, anti-GAD65 antibodies have been reported to be present in up to 80% of DM1 patients [14]. While the epitopes for anti-GAD65 vary between SPS and DM1 patients, 30% of SPS patients have DM1 [11]. This can further support the diagnosis of SPS if a patient presents with DM1. In addition to being a part of this overlapping group of patients, our patient also displayed the characteristic clinical course whereby DM1 diagnosis occurred prior to the onset of SPS. While DM1 typically has childhood onset before the age of 14, our patient presented with late-onset DM1, being diagnosed at the age of 28; anti-GAD65 has been interpreted to be a biomarker for DM1 [4]. 

Anti-GAD65 and refractory epilepsy 

Epilepsy is one of the core neurological diseases associated with anti-GAD65 [8,12,15]. In fact, the onset of epilepsy occurs earlier than the onset of other anti-GAD65 disease associations such as SPS and cerebellar ataxia, and often presents concurrently with syndromes such as SPS [15]. However, one study found that refractory epilepsy was more likely to be found in isolation than along with SPS [15]. Further, another study found that epilepsy in SPS is often well controlled [8]. Indeed, our patient has experienced refractory epilepsy - trying different medications - prior to the diagnosis of SPS.

## Conclusions

The diagnosis of SPS can be challenging as the range of symptoms can be broad and diagnostic criteria remain vague. Moreover, SPS is a progressive disease, as symptoms without therapy worsen over time: increased stiffness and falls, and decreased ability to carry out daily activities of living. Thus establishing diagnostic criteria such as high titers of anti-GAD65, and the presence of other autoimmune conditions such as DM1 and anti-GAD65 diseases such as epilepsy can be useful in the timely diagnosis of SPS. However, further areas of research may include ascertaining a threshold for anti-GAD65 titers and establishing a strong association between concurrent diseases: DM1, SPS, and epilepsy.
